# Collagen isolation and characterization from *Sardinella longiceps*

**DOI:** 10.5455/javar.2021.h560

**Published:** 2021-11-15

**Authors:** Sobanalakshmi Srinivasan, Brindha Durairaj

**Affiliations:** Department of Biochemistry, PSG College of Arts and Science, Coimbatore, India

**Keywords:** Isolation of fish collagen, Sardinella longiceps, acid-soluble collagen, pepsin-soluble collagen characterization

## Abstract

**Objective:**

Collagen is a fibrous protein that is primarily used in the pharmaceutical and biomedical industries. This study isolates and characterizes type-1 collagen from *Sardine longiceps* (scales, skin, and muscle).

**Materials and Methods:**

Collagen was isolated from *S. longiceps* using two methods: acid-solubilized collagen and pepsin-solubilized collagen. Sodium dodecyl sulfate–polyacrylamide gel electrophoresis (SDS–PAGE) was used to estimate the molecular weight of isolated collagen. Ultraviolet (UV)-visible spectrophotometry analysis was used to confirm the collagen extracted (type-I collagen). The functional groups of isolated collagens were identified using fourier transform infrared (FTIR) analysis. The X-ray diffraction (XRD) technique was used to investigate the crystallinity of isolated collagen. The high-pressure liquid chromatography (HPLC) technique was used to study the amino acid composition.

**Results:**

SDS–PAGE of *S. longiceps *revealed molecular weights ranging from 116 kDa for α-2 to 97 kDa for α-1. UV-visible spectra showed an absorbance value below 300 nm, and the results confirmed type-I collagen. FTIR showed major functional groups like amide A, B, I, II, and III. XRD determined the crystallinity of isolated collagen. The HPLC results showed the presence of higher glycine content, followed by proline and hydroxyl proline in the extracted collagen.

**Conclusion:**

The overall study confirmed that fish waste materials (scales, skin, and muscles) could be used as an alternative source for collagen.

## Introduction

Collagen is an essential extracellular protein in vertebrates and is abundantly present in the skin and muscles. In mammals, collagen can account for up to 30% of the total protein [1]. Collagen possesses low antigenic, anti-inflammatory, biocompatible, and biodegradable properties. There are 28 types of collagen identified, and among these type-I collagen is widely present in connective tissues.

Collagen protein has a triple helical structure composed of three polypeptide chains. The main features of the helix formation are high glycine content and low proline and hydroxyproline content. Collagen is mainly produced from animal skin, muscles, and bones, such as bovine and porcine. Animal collagen is primarily used in industrial settings. Outbreaks of animal collagen-transmitting illnesses, including bovine spongiform encephalopathy, transmissible spongiform encephalopathy, and foot and mouth disease, have limited the use of animal collagen owing to the risk of disease transmission to humans. In such circumstances, the best alternative new sources of collagen are found in marine fishes, sponges, freshwater fishes, scallop mantle, and oysters.

Fish waste material is an effective alternative source for collagen and has no disease transmission to humans because they are not linked with pathogens. Marine collagen has wide applications in the pharmaceutical, biomedical, and cosmetics industries [2,3].

Seafood processors’ disposal of fish processing waste materials (scales, skin, and muscles) poses a potential risk to the environment and public health. However, utilizing fish waste material to isolate collagen will reduce improper dumping of this waste into landfills. Collagen is derived from the commercially accessible fish, *Sardinella longiceps*, which humans consume. Previous reports suggest that collagen isolated from fish possesses wound healing, anti-larvicidal, and anti-aging properties [2,4]. The objective of this study was to isolate and characterize type-1 collagen from *Sardine longiceps* (scales, skin, and muscle).

## Materials and Methods

### Sample collection

**Figure 1.**
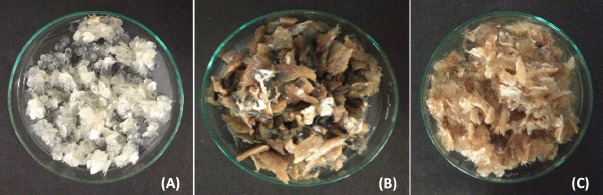
Sample of sardine (A) scales, (B) skin, and (C) muscles.

Indian sardine (*S. longiceps*) scales, skin, and muscles were collected from a local fish market in Coimbatore, Tamil Nadu. The fish scales, skin, and muscles were chopped into small pieces and rinsed with water (Fig. 1). The samples are cleaned before being utilized to isolate collagen.

### Sample preparation

The fish scales, skin, and muscles were washed twice in a 10% NaCl solution and stirred for 24 h to eliminate any excessive proteins. All the processes were carried out at a temperature of 4°C. The fish scales are demineralized for 90 min in a 0.4 M HCl solution to extract soluble collagen. The scales, skin, and muscles of the fish (*S. longiceps*) were washed and cut into small pieces (about 5 mm) with sterile scissors to isolate the collagen process. The skin and muscles of the fish were immersed in a 10% butanol solution and constantly stirred to remove the fat content.

### Isolation of collagen

#### Acid-solubilized collagen (ASC) method

All processes are carried out at a temperature of 4°C. The scales, skin, and muscles of sardine fish were removed for 4 days using 0.5 M acetic acid. The extract was collected into a conical flask and centrifuged at 4,000 rpm for 30 min using a cotton cloth. The supernatant was then salted out by adding 0.7 M NaCl and stored at 4°C overnight. Precipitation was used to isolate the protein in this stage. Centrifugation at 4,000 rpm for 30 min separated the precipitated collagen. The collagen residues were dialyzed in 100 volumes of 0.1 M acetic acid, followed by distilled water, after being dissolved in 0.5 M acetic acid at a ratio of 1:10 (w/v). After that, a freeze dryer was used to lyophilize the dialyzed material. The separated collagen was kept for further research.

#### Pepsin-solubilized collagen (PSC) method

Pepsin-soluble collagen was isolated from the scales, skin, and muscles obtained following acid isolation. The processed samples were soaked in a 1:15 (w/v) ratio in 0.5 M acetic acid containing 1.5% pepsin. The combinations were maintained at 4°C for 4 days before being filtered using filter paper [3]. The filtrate was collected in a conical flask and treated with the same precipitation and dialysis procedure as in ASC isolation. Based on the dry weight, the ASC and PSC yields were estimated using the following formula:


$$\mathrm{Yield}\;\mathrm{of}\;\mathrm{collagen}\;(\mathrm{wet})\;(\mathrm{\%)}=\frac{\mathrm{weight}\;\mathrm{of}\;\mathrm{lyophilized}\;\mathrm{collagen}\;(\mathrm{gm})}{\mathrm{Weight}\;\mathrm{of}\;\mathrm{wet}\;\mathrm{fish}\;\mathrm{waste}\;\mathrm{materials}\;(\mathrm{gm})}\times 10$$

#### Quantification of collagen protein by Lowry’s method

Lowry’s technique was used to measure the protein content of extracted collagen (ASC and PSC) using bovine serum albumin as a reference [4].

### Sodium dodecyl sulfate–polyacrylamide gel electrophoresis (SDS–PAGE)

SDS–PAGE –was used to identify the molecular weight of isolated collagen, and gel electrophoresis was conducted. Using a Mini-Protean II electrophoresis machine, the SDS–PAGE was conducted according to that described previously [5]. A mixture of glycine and Tris buffer made up the running buffer. The protein was dyed with Coomassie blue. 4% polyacrylamide was used in the stacking gels, while 7.5% polyacrylamide was used in the separating gels [6]. The sample buffer was combined with the extracted protein sample in a 1:1 ratio (0.5 M Tris HCl, pH 6.8, containing 10% SDS and 100% glycerol, 8% b-ME, and bromophenol blue 0.1%). The extracted collagen sample was heated for 5–10 min at 100°C. After chilling, the samples were placed onto polyacrylamide gels with 7.5% running gel and 4% stacking gel. The protein migration was initiated at 80 V with a continuous current until the collagen peptides went through the stacking gel, then increased to 150 V until the resolving gel reached the endpoint. The gel was stained with 0.05% (w/v) Coomassie blue R-250 in 15% (v/v) methanol and 5% (v/v) acetic acid after the electrophoresis procedure was completed, and then destained with 30% (v/v) methanol and 10% (v/v) acetic acid. The molecular weights of isolated collagen from fish waste products were determined using conventional protein markers.

### Characterization of collagen

#### Ultraviolet (UV)–Vis spectroscopy

The scales, skin, and muscles of sardines (*S. longiceps*) were dissolved in 0.5 M acetic acid to a concentration of 2 gm/l of ASC and PSC. The UV-visible spectra were examined using a UV-vis spectrophotometer.

#### Fourier transform infrared (FTIR) spectroscopy

FTIR spectra were obtained using a Shimadzu 8201 PC FTIR analyzer to establish that the separated collagen samples were type-1 collagen (ASC and PSC). The spectral range between 4,000 and 400 cm^−1^ was studied, and spectra were acquired using the KBr disk technique. 

#### X-ray diffraction (XRD)

The XRD pattern of isolated collagen samples was recorded using an X-ray (XPERT-PRO) operating at a voltage of 40 kV and a current of 30 mA with Cu Ka radiation (*k* = 1.54060 Å). The XRD pattern was recorded in a fixed time mode at room temperature in the 2 h range of 100–800.

#### Amino acid analysis

High-pressure liquid chromatography (HPLC) was used to determine the amino acid composition of the extracted collagen [7]. The ASC and PSC samples were dissolved in 6 N HCl for 24 h at 110°C (hydrolysis in a boiling water bath). The samples were centrifuged for 20 min at 3,500 rpm after 24 h. For the neutralization procedure, the supernatant was filtered and 1 N NaOH was added. After pre-column derivatization with solution O-phthaldialdehyde, HPLC analysis was carried out at a range of 340–280 nm wavelength. ASC and PSC amino acid compositions were expressed as a proportion of protein.

## Results

### Collagen yields

**Figure 2.**
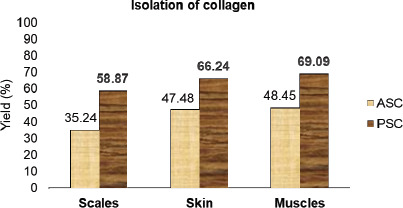
Yield (%) of isolated collagen from scales, skin, and muscle of sardine (ASC, acid-soluble collagen; PSC pepsin-soluble collagen).

On a wet basis, 100 gm of scales, 250 gm of skin, and 300 gm of muscles were used to extract collagen from sardine fish. After being lyophilized, PSC was obtained as a pale gray-colored soft fibrous substance, while ASC was obtained as a colorless soft fibrous substance. ASC solubilized collagen yield was lower than the PSC yield. The yield obtained from ASC was 35.24% for scales, 47.48% for skin, and 48.45% for muscles, compared with the yield of PSC, which was 58.87% for scales, 66.24% for skin, and 69.09% for muscles. The collagen yield was increased after adding the pepsin enzyme with acetic acid, which completely solubilized the collagen (Fig. 2).

### SDS–PAGE

**Figure 3.**
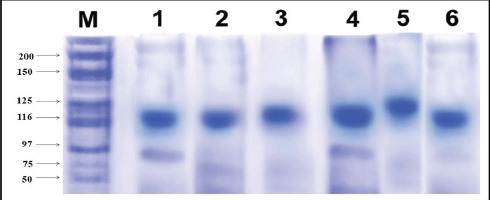
SDS–PAGE pattern of sardine scales’ collagen. Lane 1: protein marker (M); Lane 2: -ASC (scales); Lane 3: -PSC (scales); Lane 4: ASC- (skin); Lane 5: ASC- (muscles); and Lane 6: PSC- (muscles).

SDS–PAGE was carried out to determine the molecular weight of isolated collagen from sardine scales, skin, and muscles by two different isolation methods (ASC and PSC). The isolated collagen structure showed one α-band and two α-chains (α-1 and α-2) with the same mobility. The band showed a type-1 collagen pattern (Fig. 3). The band intensity of PSC was higher than ASC. The molecular weight of the isolated collagen was 116 kDa for α-2 and 97 kDa for the α-1 chain, and molecular weight markers showed that the isolated collagen from fish waste materials was type I.

### UV–Vis spectroscopy

**Figure 4.**
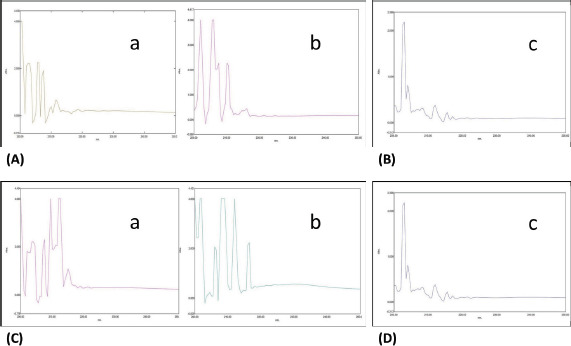
(A) UV spectra of ASC- from scales (a) and skin (b) of sardine fish; (B) UV spectra of ASC- from muscles (c) of sardine; (C) UV spectra of PSC- from scales (a) and skin (b) of sardine; and (D) UV spectra of PSC- from muscles of sardine.

The collagen isolated from sardines (scales, skin, and muscles) showed an absorbance value below 300 nm (Table 1); the bands are labeled in Figure 4 a and b (ASC) and c and d (PSC). The tyrosine and phenylalanine amino acids are sensitive chromophores absorbing the UV-visible spectra below 300 nm [7]. Hence, the extracted protein confirmed the presence of collagen.

### FTIR spectroscopy

**Figure 5.**
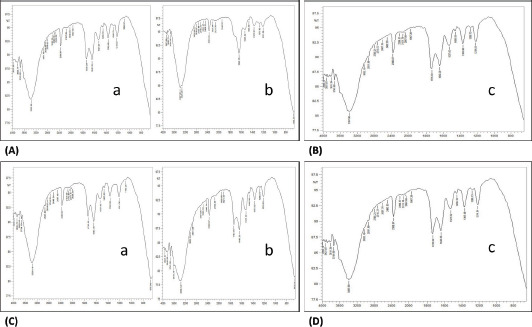
(A) FTIR spectra of ASC- from scales (a) and skin (b) of sardine fish; (B) FTIR spectra of ASC- from muscles (c) of sardine fish; (C) FTIR spectra of PSC- from scales (a) and skin (b) of sardine fish; and (D) FTIR spectra of PSC- from muscles (c) of sardine fish.

The results of FTIR spectra of ASC and PSC isolated from sardine scales, skin, and muscles observed the presence of functional groups (4,000–400 cm^−1^). The FTIR spectra of ASC- and PSC- isolated from sardine fish showed several bands, such as proteins, carbohydrates, lipid, and symmetric and asymmetric (NH, C–H, C = O, CH_3_, CH_2_, and OH) groups and amide A, B, I, II, and III stretching bands of proteins. The bands are shown in Figure 5 a and b (ASC) and c and d (PSC). The peak range of ASC and PSC is presented in Table 2.

### X-ray diffraction (XRD)

**Figure 6.**
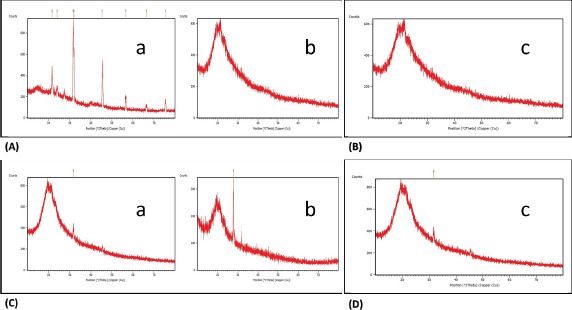
(A) XRD pattern of ASC- from scales (a) and skin (b) of sardine fish; (B) XRD pattern of ASC- from muscles (c) of sardine fish; (C) XRD pattern of PSC- from scales (a) and skin (b) of sardine fish; and (D) XRD pattern of PSC- from muscles (c) of sardine fish.

The XRD pattern determines the collagen fibril dispersion. The XRD pattern of ASC and PSC from sardines is shown in Figure 6 a–d. The XRD pattern of sardine waste materials (scales, skin, and muscles) is presented in Table 3. XRD revealed that the extracted protein showed consecutive morphology. Therefore, it is confirmed that the extracted collagen exhibited a triple helix structure.

### Amino acid analysis

The analysis of the amino acid composition of ASC and PSC from sardine fish waste material was carried out using HPLC (Table 4). Collagen contains a high concentration of glycine amino acid, followed by proline and hydroxyproline. The highest glycine content (18.5–20.1) is found in collagen isolated from sardine fish waste materials, followed by proline (5.5) and hydroxyproline (5.4).

## Discussion

The yield of collagen isolated from *S. longiceps *was 43.7% for ASC and 63.7% for PSC. These present results are comparable with those of Nagai et al. [8], who reported the same in marine fishes like yellow sea bream (40%) and brown-backed toadfish (54.3%). Hence, sardine fish scales, skin, and muscles were an alternative source of collagen for the industrial, pharmaceutical, and cosmetic sectors [6,9,10]. The SDS–PAGE was analyzed based on the molecular weight. The banding pattern of ASC and PSC was observed at type-I collagen molecular weight. SDS–PAGE was compared with leather jacket fish scale collagen [11,12].

**Table 1.** UV peak range of the extracted collagen (ASC and PSC) from sardine fish.

Fish waste materials	ASC (nm)	PSC (nm)
Scales	210	230
Skin	200	240
Muscles	210	250

This study suggests that both ASC and PSC isolated from sardine fish processing materials are probably type-I collagen. The intermolecular crosslinks of collagen were observed in the ASC and PSC isolation methods. These findings were compared with Liu et al. [13], who reported that the collagen isolated from fish scales, skin, and muscles of snakehead fish, rohu, and *Catla catla* is crosslinked by intra- and interbonding. Similarly, Kittiphattanabawon et al. [14] obtained the same results as leather jacket and *Cirrhinus mrigala*. The UV-spectrum peak observed from the ASC and PSC displayed different peak ranges between 206 and 215 nm, which confirmed the triple helix. The absorption peak of collagen correlated to the reported data of the channel catfish, the skin of walleye Pollock [15–17], and freshwater fish.

FTIR studies revealed the location of amide I, II, III, A, and B, illustrating the configuration of a polypeptide. The present observation is comparable with the data reported in eel fish *Evenchelys macrura* skin. The XRD results of ASC and PSC were compared with the findings of Kittiphattanabawon et al. [18], who reported on the XRD pattern of carp fish scales collagen at the angles (2 h) of 7.43 and 19.78. This characterization is also congruent with Ali et al. [19], who obtained a similar result. The HPLC results of ASC and PSC obtained from sardine scale, skin, and muscles were observed to contain essential and non-essential amino acids. These findings relate to those of other researchers [20–22], who have stated that a similar amino acid composition was obtained for collagen extracted from *C. catla* and bigeye snapper fish.

**Table 2.** FTIR peak range of extracted collagen (ASC and PSC) from sardine fish.

Fish waste materials	Properties	ASC (nm)	PSC (nm)	Peak region assignment
Scales	Amide-A	3,363	3,356	N–H bond (stretching of proteins)
	Amide-B	2,931	2,931	CH2—asymmetric stretch
	Amide-I	1,643	1,643	C = O bond (stretching of proteins)
	Amide-II	1,527	1,530	N–H bond (Bending/C–N stretching of proteins)
	Amide-III	1,219	1,211	C–O bond (stretch/NH bond coupled with C–N stretch)
Skin	Amide-A	3,379	3,387	N–H bond (stretching of proteins)
	Amide-B	2,931	2,924	CH2—asymmetric stretch
	Amide-I	1,643	1,643	C = O bond (stretching of proteins)
	Amide-II	1,535	1,535	N–H bond (Bending/C–N stretching of proteins)
	Amide-III	1,211	1,211	C–O bond (stretch/NH bond coupled with C–N stretch)
Muscles	Amide-A	3,387	3,356	N–H bond (stretching of proteins)
	Amide-B	2,353	2,931	CH2—asymmetric stretch
	Amide-I	1,643	1,643	C = O bond (stretching of proteins)
	Amide-II	1,535	1,527	N–H bond (Bending/C–N stretching of proteins)
	Amide-III	1,211	1,219	C–O bond (stretch/NH bond coupled with C–N stretch)

**Table 3.** XRD bands displayed by collagen protein (ASC and PSC) of sardine fish.

Fish Waste Materials	ASC	PSC
Scales	21°	24°
Skin	27°	25°
Muscles	28°	23°

**Table 4.** Amino acid composition of scales, skin, and muscles collagen of sardine.

Amino acid	Scales (%)	Skin (%)	Muscles (%)
Hydroxyproline	4.4	5.2	5.8
Aspartic acid	1.0	3.2	6.1
Threonine	5.4	3.1	6.6
Serine	4.2	4.6	5.2
Glutamic acid	9.7	10.1	10.1
Proline	6.1	6.1	6.1
Glycine	19.1	19.3	20.4
Alanine	1.4	1.8	2.0
Valine	3.7	3.7	5.2
Methionine	1.1	1.1	0.6
Isoleucine	1.4	1.6	1.7
Leucine	4.4	4.4	5.4
Tyrosine	3.4	4.4	4.6
Phenylalanine	0.3	0.5	1.0
Lysine	2.0	3.4	4.1
Histidine	3.0	3.3	3.5
Arginine	9.0	9.0	9.0
Total	100	100	100
Amino acids	12.4	12.6	12.8

## Conclusion

The extracted collagen was type 1, as determined by two different isolation techniques (ASC and PSC). It has a wide range of applications in the biomedical and pharmaceutical sectors. Isolated collagen from fish waste material is the best alternative source for producing high value-added products, but it also helps reduce environmental disposal.

## List of abbreviations

ASC, Acid-soluble collagen; FTIR, Fourier transform infrared; HPLC, High-pressure liquid chromatography; PSC, Pepsin-soluble collagen; SDS–PAGE, Sodium dodecyl sulfate–polyacrylamide gel electrophoresis; UV, Ultraviolet-visible; XRD, X-ray diffraction.
